# Comparison of Pregnancy Preferences Preceding vs Year 1 of the COVID-19 Pandemic

**DOI:** 10.1001/jamanetworkopen.2022.20093

**Published:** 2022-07-05

**Authors:** Corinne H. Rocca, Miriam Parra, Isabel Muñoz, Diana G. Foster, W. John Boscardin, Lauren J. Ralph

**Affiliations:** 1 Bixby Center for Global Reproductive Health, Advancing New Standards in Reproductive Health, School of Medicine, Department of Obstetrics, Gynecology, and Reproductive Sciences, University of California, San Francisco, Oakland; 2 School of Nursing, University of California, San Francisco; 3 School of Public Health, Division of Epidemiology, University of California, Berkeley; 4 School of Medicine, Department of Medicine, University of California, San Francisco; 5 School of Medicine, Department of Epidemiology and Biostatistics, University of California, San Francisco

## Abstract

**Importance:**

Understanding how the COVID-19 pandemic affected people’s desire to avoid pregnancy is essential for interpreting the pandemic’s associations with access to reproductive health care and reproductive autonomy. Early research is largely cross-sectional and relies on people’s own evaluations of how their desires changed.

**Objective:**

To investigate longitudinal changes in pregnancy desires during the year before and the first year of the COVID-19 pandemic.

**Design, Setting, and Participants:**

In this cohort study, participants reported their pregnancy preferences at baseline and quarterly for up to 18 months between March 2019 and March 2021. An interrupted time series analysis with mixed-effects segmented linear regression was used to examine population-averaged time trends. People were recruited from 7 primary and reproductive health care facilities in Arizona, New Mexico, and Texas. Participants were sexually active, pregnancy-capable people aged 15 to 34 years who were not pregnant or sterilized. Data analysis was performed from September 2021 to January 2022.

**Exposures:**

Continuous time, with knots at the onset of the first (July 1, 2020, summer surge) and second (November 1, 2020, fall surge) COVID-19 cases surges in the Southwest.

**Main Outcomes and Measures:**

Preferences around potential pregnancy in the next 3 months, measured using the validated Desire to Avoid Pregnancy (DAP) scale (range, 0-4, with 4 indicating a higher desire to avoid pregnancy).

**Results:**

The 627 participants in the analytical sample had a mean (SD) age of 24.9 (4.9) years; 320 (51.0%) identified as Latinx and 180 (28.7%) as White. Over the year before the first case surge in the US Southwest in summer 2020, population-averaged DAP scores decreased steadily over time (−0.06 point per quarter; 95% CI, −0.07 to −0.04 point per quarter; *P* < .001). During the summer 2020 surge, DAP scores stopped declining (0.05 point per quarter; 95% CI, −0.03 to 0.13 point per quarter; change in slope, *P* < .001). During the fall 2020 surge, however, DAP scores declined again at −0.11 point per quarter (95% CI, −0.26 to 0.04 point per quarter; change in slope, *P* = .10). Participants aged 15 to 24 years and those who were nulliparous and primiparous experienced greater declines in DAP score before the summer surge, and greater reversals of decline between summer and fall 2020, than did those who were aged 25 to 34 years and multiparous.

**Conclusions and Relevance:**

These findings suggest that the COVID-19 pandemic onset was associated with the stalling of a prior trend toward greater desire for pregnancy over time, particularly for people earlier in their reproductive lives.

## Key Points

**Question:**

How did people’s pregnancy preferences change over the year before and the first year of the COVID-19 pandemic?

**Findings:**

In a cohort study of 627 participants aged 15 to 34 years in the US Southwest, the summer 2020 COVID-19 case surge was associated with a significant short-term curtailing of a pre-COVID-19 trend toward greater desire for pregnancy. Trends were most pronounced among younger nulliparous and primiparous participants.

**Meaning:**

Given that disruptive events can increase the desire to prevent or postpone pregnancy, expanded contraceptive and abortion care models like pharmacy access will be important to reproductive autonomy during future disruptions.

## Teaser

This cohort study investigates longitudinal changes in pregnancy desires during the year before and the first year of the COVID-19 pandemic.

## Introduction

The COVID-19 pandemic has resulted in widespread disruption of people’s social and economic lives, including their sexual and reproductive health. This social disturbance, economic insecurity, and strain on health systems may have compromised access to contraceptive care, fertility treatment, abortion, and autonomy over sexual behavior and may have changed people’s desires around pregnancy and childbearing.^1,2,3,4,5^ Interpreting the degree to which any observed changes in individuals’ reproductive health, including in pregnancy rates, birth rates and intervals, and abortion rates, are due to modified childbearing desires vs people’s compromised ability to attain their childbearing desires requires understanding how pregnancy preferences changed during the pandemic.

A growing body of cross-sectional, usually internet-based, survey research has found that reports of modified pregnancy desires were common early in the pandemic. Conducted in the US^6,7,8^ and other countries in the Northern Hemisphere,^9,10,11,12,13^ these surveys have broadly found up to one-half of respondents reporting that their desire for pregnancy declined (or that they wanted to postpone pregnancy), citing health risks, financial concerns, loss of income, and sense of uncertainty about the future. At the same time, these studies have found smaller proportions of respondents reporting increased desire for pregnancy because of the pandemic, owing to wanting change and positivity in their life, lower opportunity costs of taking time off work for child-rearing, and a recalibration of priorities.

Despite these findings, robust evidence on how the COVID-19 pandemic affected pregnancy desires at the population level remains sparse because of important methodological limitations. First, virtually all research to date has been cross-sectional and has relied on respondents’ own evaluations of how their pregnancy desires had changed because of the pandemic, rather than comparing prepandemic preferences with those measured during the pandemic. Reproductive health experts have increasingly recognized that many individuals do not hold clear pregnancy desires or intentions, especially if their life circumstances have not prompted them to form an explicit intention.^14,15,16^ As a result, people may not be fully aware of their preferences, know when changes happened, or attribute changes specifically to the pandemic. To date, we have lacked prospective data on pregnancy desires both before the pandemic and as the pandemic evolved over many months.

Furthermore, until recently,^14^ research has lacked rigorous measurement instruments to capture the full range of feelings people have about pregnancy,^17,18^ relying instead on simple assessments of trying or not trying, or wanting more or wanting less. Such categories do not comprehensively capture the preferences people have about a potential pregnancy, nor do they account for ambivalence or uncertainty.^19,20^

In this cohort study, we used longitudinal data and an interrupted time series approach to examine changes in people’s pregnancy desires over the years before (March 2019 to March 2020) and after (March 2020 to March 2021) the onset of the COVID-19 pandemic. We hypothesized that, on the population level, there would be an increase in preference to avoid pregnancy with the onset of the shelter-in-place mandate and during the two 2020 surges in COVID-19 cases. To our knowledge, this study is the first to use repeated measurements among the same individuals over time, over a year before and a year during the pandemic, to investigate changes in pregnancy preferences.

## Methods

### Study Procedures and Participants

The study protocol was approved by the University of California, San Francisco’s institutional review board. We followed the Strengthening the Reporting of Observational Studies in Epidemiology (STROBE) reporting guideline for cohort studies.

Data were drawn from the first 2 years of the Attitudes and Decisions After Pregnancy Study, an ongoing observational cohort study examining pregnancy preferences, pregnancy decision-making, and the impact of pregnancy on people’s health and lives. Participants were recruited in the waiting rooms of 7 primary care and reproductive health facilities in Arizona, New Mexico, and Texas between March 16, 2019, and February 16, 2020. To be eligible, patients had to read and speak English or Spanish and be aged 15 to 34 years, with parental consent required for minors aged 15 to 17 years if required by state law and the facility to obtain the care they were seeking. Patients had to have the capacity for pregnancy (eg, a uterus), have been sexually active with a person with sperm within 3 months, and live in a study state or bordering state. Patients were ineligible if they were sterilized, had an intrauterine device or subdermal implant placed, or were currently pregnant, unless recruited at an abortion appointment.

Eligible patients who agreed to participate completed written informed consent with a trained research assistant and received a link to an online survey to be completed within 2 weeks. Participants could opt to complete their surveys via telephone with a research assistant. Baseline surveys asked about participants’ pregnancy preferences and sociodemographic and partnership characteristics. Participants were followed for a minimum of 1 year and were asked to complete surveys every 6 weeks about new pregnancies and quarterly on a set of reproductive health indicators. A subset of participants (approximately 1 in 6) was monitored for up to 2 additional years for a separate component of the study. Participants were remunerated with a $50 gift card for completing the baseline survey, $20 for each quarterly follow-up survey, and $5 for interim pregnancy check-ins.

### Measures

Our dependent variable of interest was preferences about a potential pregnancy measured with the Desire to Avoid Pregnancy (DAP) Scale.^14^ The DAP Scale is a validated, 14-item instrument that measures preferences about pregnancy in the next 3 months and childbearing within the next year across 3 domains: cognitive desires, affective feelings, and anticipated consequences. The DAP is unique conceptually in that it does not assume individuals hold clear intentions and allows people’s preferences to be ambivalent and underspecified. It purposefully asks about preferences within a short time frame, acknowledging that many people do not formulate long-term plans about childbearing and that preferences change with changing life circumstances. Response options for each item are a 5-point scale (strongly agree, agree, neither agree nor disagree, disagree, and strongly disagree); responses are averaged for a final DAP score of 0 to 4, with 4 representing a higher desire to avoid pregnancy and 0 indicating greater openness to pregnancy or, for some, desire for pregnancy.

The independent variable was continuous calendar time, from March 16, 2019, through March 15, 2021. Time coefficients are presented in quarters (eg, 3 months). We included covariables identified a priori as factors associated with pregnancy preferences, including age, participant-identified race and ethnicity, parity, partnership and cohabitation status, and experiencing food insecurity. Race and ethnicity were included to capture the sociocultural norms and life experiences (eg, racism) that can influence pregnancy preferences. Participants could choose from prespecified options and/or provide their own response. Those indicating multiple races or ethnicities were subsequently asked the race or ethnicity with which they most identified. We used food insecurity as an indicator of socioeconomic status because household income is often misreported and is often unknown among adolescents.

### Statistical Analysis

Data analysis was performed from September 2021 to January 2022. Analyses included participants enrolled into the study through February 16, 2020, each contributing at least 1 observation before the onset of the COVID-19 shelter-in-place mandate and a maximum of 18 months of observation through March 15, 2021. Participants experiencing pregnancy were included in analyses until the pregnancy was reported. We described numbers of participants screened, eligible, and completing the baseline survey during the year before the onset of COVID-19. We compared age, race and ethnicity, and screening language between eligible patients who completed baseline vs those who did not using mixed-effects models that account for site clustering.

We conducted segmented regression analysis of interrupted time series data to examine population-averaged trajectories in DAP scores from March 16, 2019, to March 15, 2021.^21,22^ Our modeling approach was multivariable mixed-effects linear regression. Models included calendar time, modeled continuously as a linear spline with knot locations chosen as described later and with possible discontinuities (jumps) at the knots; possible interactions of the time trajectory with sociodemographic factors as described later; and random effects for participant-specific intercept and slope to allow for unique trajectories for each participant, which improved models’ fit over random intercept only models, based on log likelihood ratio tests, with significance set at *P* < .05. We used Centers for Disease Control and Prevention data to identify relevant COVID-19 dates to serve as hypothesized change points (eFigure in the Supplement).^23^ For knot locations in the linear spline, we considered (1) the onset of shelter-in-place mandates in study states (April 1, 2020); (2) 1 month into the first surge in cases in the study region following the lifting of shelter-in-place orders in summer 2020 (July 1, 2020); and (3) 1 month into the large second surge in cases in fall 2020 (November 1, 2020). We confirmed we had not missed notable change points by using data visualization, fitting models with fixed effects for month and quarter, as well as examining Harrell recommended knot locations.^24^

We first fit a full segmented regression model that included the 3 hypothesized change points. We examined adding discontinuities (jumps) in DAP scores at the change points; however, we excluded these parameters because we found no evidence of abrupt changes at the change points at the *P* < .10 level. Our final model included 2 change points, during the summer and fall surges, as no change in slope was observed at the onset of shelter-in-place. Time coefficients can be interpreted as mean change in DAP score over 3 months. We conducted complete case analyses because missing data were minimal (<3% for any variable).

To explore whether the degree of change in DAP scores differed by sociodemographic factors, we fit companion models that included interactions for factors we hypothesized post hoc might affect how people’s pregnancy preferences change in response to COVID-19. We included age group (15-24 vs 25-34 years), parity (nulliparous and primiparous vs multiparous), partnership status (main partner vs not), and food insecurity (yes vs no). We considered an interaction to be present if a joint test of time-by-factor interaction terms was significant at *P* < .10, indicating nonparallel trajectories. Analyses were conducted with Stata statistical software version 15 (StataCorp); results are reported at the 2-sided *P* < .05 significance level.

## Results

### Participant Characteristics

The 627 participants had a mean (SD) age of 24.9 (4.9) years at baseline (Table 1). Forty-six participants (7.3%) identified as Black, 320 (51.0%) were Latinx, 180 (28.7%) were White, and 81 (12.9%) were multiracial or identified with another race or ethnicity (eg, Alaska Native, American Indian, Asian, Middle Eastern, Native Hawaiian, North African, and Pacific Islander). Two hundred eighty-six participants (45.8%) were nulliparous, 134 (21.5%) were primiparous, and 204 (32.7%) were multiparous. Three hundred fifteen (50.3%) were living with a main romantic partner, and 231 (37.8%) had experienced food insecurity in the prior month.

**Table 1.** Baseline Participant Characteristics

Characteristic	Participants, No. (%) (N = 627)^a^
Age, mean (SD) [range], y^b^	24.9 (4.9) [15-35]
Age group, y	
15-19	102 (16.3)
20-24	203 (32.4)
25-29	193 (30.8)
30-34	129 (20.6)
Race and ethnicity	
Black	46 (7.3)
Latinx	320 (51.0)
White	180 (28.7)
Multiracial or other^c^	81 (12.9)
Parity (n = 624)	
0, nulliparous	286 (45.8)
1, primiparous	134 (21.5)
2, multiparous	118 (18.9)
≥3, multiparous	86 (13.8)
Partnership status (n = 626)	
Has a main partner, living together	315 (50.3)
Has a main partner, not living together	204 (32.6)
Has no main partner	107 (17.1)
Food insecurity in last month (n = 611)	
Yes	231 (37.8)
No	380 (62.2)
Reason for clinic visit (n = 613)	
Contraceptive care	257 (41.9)
Abortion	95 (15.5)
Other reproductive health care	203 (33.1)
Nonreproductive primary care	58 (9.5)
Current contraceptive method	
None	162 (25.8)
Natural or withdrawal	69 (11.0)
Condom	115 (18.3)
Short-acting hormonal	241 (38.4)
Intrauterine device or implant^d^	40 (6.4)

^a^ Percentages may not add to 100% because of rounding.

^b^ The sample includes 4 participants determined after enrollment to be aged 35 years by their baseline survey.

^c^ Other includes Alaska Native, American Indian, Asian, Middle Eastern, Native Hawaiian, North African, Pacific Islander.

^d^ Participants had an intrauterine device or implant placed between eligibility screening and completing the baseline survey.

### Enrollment

**Figure 1.**
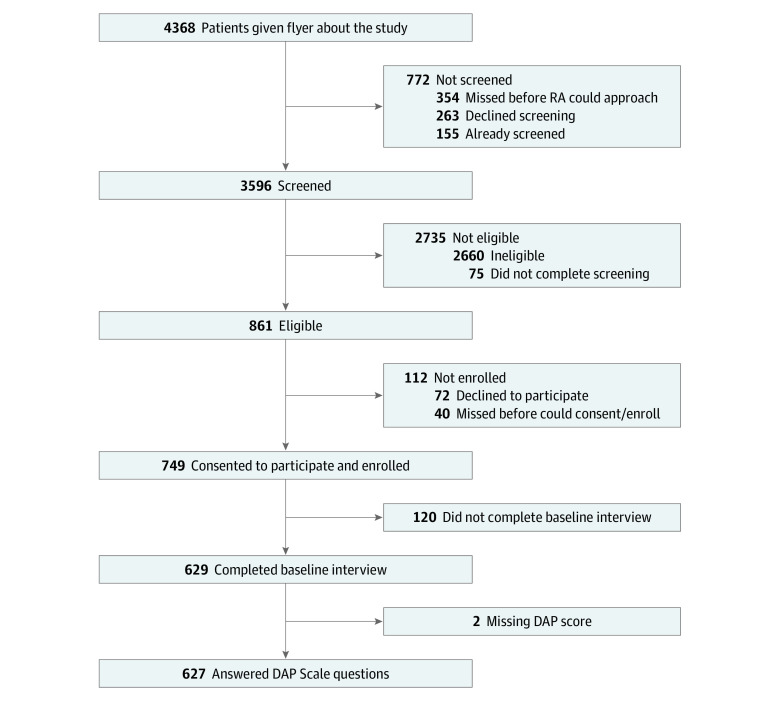
Flowchart of Enrollment Into the Attitudes and Decisions After Pregnancy Study Before March 15, 2020 The flowchart presents the numbers of participants screened, eligible, consenting to participate, and completing the baseline interview of the study. DAP indicates Desire to Avoid Pregnancy Scale; RA, research assistant.

During recruitment, 4368 potential participants entered clinic waiting rooms, 3596 of whom completed eligibility screening; 861 were eligible (Figure 1). The most common reasons for ineligibility included pregnancy (1330 participants [50.5%]), age (695 participants [26.5%]), having an intrauterine device or implant (487 participants [18.6%]), no recent sexual activity (383 participants [14.9%]), and being sterilized (241 participants [9.2%]). Among eligible patients, 749 (86.6%) were enrolled. Eligible patients who completed the baseline survey did not differ from eligible patients who did not in terms of age, race and ethnicity, or language. Among the 629 participants who completed baseline, 2 did not contribute to analyses because none of their observations included a DAP score. Analyses include 2817 observations (median [IQR], 5 [5-5] observations) among 627 participants; 544 participants (86.8%) were retained at least 6 months.

### DAP Scores

Baseline DAP scores covered the full 0 to 4 range of the scale, with a mean (SD) of 2.28 (1.08), demonstrating a slight skew toward greater desire to avoid pregnancy (median [IQR] score, 2.36 [1.50-3.14]). Three hundred eighty-six participants (61.6%) disagreed or strongly disagreed they wanted to have a baby within the next year, 202 (32.2%) agreed or strongly agreed it would be hard for them to manage raising a child if they had one in the next year, and 132 (21.1%) agreed or strongly agreed that thinking about pregnancy in the next 3 months made them feel excited (eTable in the Supplement).

### Segmented Regression

**Figure 2.**
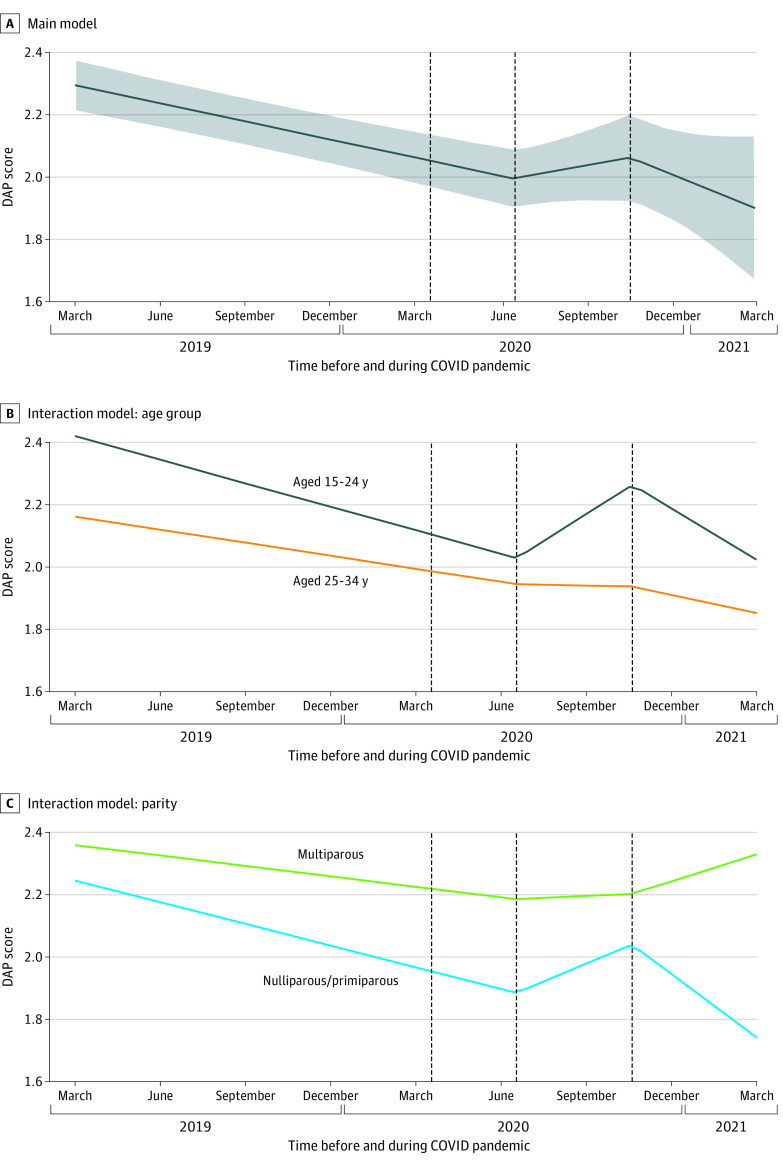
Trajectory of Desire to Avoid Pregnancy (DAP) Scale Scores Before and After the Onset of the COVID-19 Pandemic Graphs show population-averaged DAP scores over the year before and year 1 of the COVID-19 pandemic. Time (in quarters) is on the x-axis; DAP score (range, 0-4, with 4 indicating higher desire to avoid pregnancy) is on the y-axis. The 3 hypothesized changes points are indicated with dashed lines (shelter-in-place [April 1, 2020], 1 month into the summer surge [July 1, 2020], and 1 month into the fall surge [November 1, 2020]). Panel A presents DAP score trajectory for the study sample, as a whole, with 95% CIs shown with shading. Panel B presents DAP score trajectories by age group from an interaction model. Panel C presents DAP score trajectories by parity from an interaction model.

Over the year before the COVID-19 pandemic onset and the first surge of cases in summer 2020 in the US Southwest (July 1, 2020), DAP scores declined 0.06 point on the 0 to 4 DAP scale per quarter (95% CI, −0.07 to −0.04 point per quarter; *P* < .001) (Table 2 and Figure 2A). During the summer surge, this decline ceased, and DAP scores began to increase slightly, reflecting a significant change in slope to 0.05 point per quarter (95% CI, −0.03 to 0.13 point per quarter; change in slope, *P* < .001). During the fall 2020 COVID-19 surge (November 1, 2020), DAP scores resumed their pre-COVID-19 downward trajectories (−0.11 point per quarter; 95% CI, −0.26 to 0.04 point per quarter; change in slope, *P* = .10). There was marked individual variation in change in DAP before (slope SD, 0.12) and after (slope SD, 0.22) the summer COVID-19 surge.

**Table 2.** Multivariable Random Slope Mixed-Effects Model of Changes in Desire to Avoid Pregnancy Score, March 16, 2019, to March 15, 2021

Main model and time^a^	Coefficient (95% CI)^b^	Slope^c^	*P* value	
			Slope differs from 0	Change in slope from prior slope
Before summer 2020 surge	−0.06 (−0.07 to −0.04)	−0.06 (−0.07 to −0.04)	<.001	Not applicable
After summer 2020 surge	0.11 (0.02 to 0.19)	0.05 (−0.03 to 0.13)	.25	<.001
After fall 2020 surge	−0.16 (−0.35 to 0.03)	−0.11 (−0.26 to 0.04)	.14	.10

^a^ Time is treated continuously; coefficients and slopes are reported in quarters (eg, per 3 months).

^b^ Model includes participant age, race and ethnicity, parity, partnership and cohabitation, food insecurity, and recruitment site. The Desire to Avoid Pregnancy score is on a range of 0 to 4 points, with 4 indicating greater desire to avoid pregnancy.

^c^ Slope between change points can be derived by summing coefficients.

The magnitude of changes in DAP scores during the study period did not differ according to partnership status or food insecurity. However, there were significant interactions with time by age group and parity. Over the 16 months before the summer 2020 surge, DAP scores declined more steeply among those aged 15 to 24 years than those aged 25 to 34 years (−0.08 point per quarter [95% CI, −0.10 to −0.05 point per quarter] vs −0.04 point per quarter [95% CI, −0.06 to −0.02 point per quarter]; *P* = .04) (Table 3 and Figure 2B). At the summer surge, DAP trajectories among those aged 15 to 24 years reversed and increased 0.18 point per quarter (95% CI, 0.06 to 0.28 point per quarter; change in slope, *P* < .001). In contrast, among those aged 25 to 34 years, the magnitude of the decline before summer 2020 attenuated to be flat (−0.01 point per quarter; 95% CI, −0.12 to 0.10 point per quarter; no significant change in slope; difference from those aged 15-24 years, *P* = .03).

**Table 3.** Age Group–by-Time and Parity-by-Time Interaction Models: Multivariable Random Slope Mixed-Effects Models of Changes in Desire to Avoid Pregnancy Score, March 16, 2019, to March 15, 2021

Interaction model	Coefficient (95% CI)^a^^,^^b^	*P* value for interaction	Joint *P* value for interactions
Age group (reference, 25-34 y)			
Age 15-24 y	0.26 (0.07 to 0.44)	NA	NA
Time before summer 2020 surge^c^	−0.04 (−0.06 to −0.02)	NA	NA
Time after summer 2020 surge^c^	0.04 (−0.08 to 0.15)	NA	NA
Time after fall 2020 surge^c^	−0.05 (−0.31 to 0.21)	NA	NA
Age 15-24 y by time interaction before summer 2020 surge^c^	−0.03 (−0.07 to −0.002)	.04	.05
Age 15-24 y by time interaction after summer 2020 surge^c^	0.21 (0.04 to 0.38)	.02	
Age 15-24 y by time interaction after fall 2020 surge^c^	−0.29 (−0.67 to 0.09)	.13	
Parity (reference, multiparous)			
Nulliparous-primiparous	−0.11 (−0.31 to 0.08)	NA	NA
Time before summer 2020 surge^c^	−0.03 (−0.06 to −0.004)	NA	NA
Time after summer 2020 surge^c^	0.04 (−0.10 to 0.19)	NA	NA
Time after fall 2020 surge^c^	0.08 (−0.25 to 0.41)	NA	NA
Nulliparous-primiparous by time interaction before summer 2020 surge^c^	−0.04 (−0.07 to −0.001)	.04	.06
Nulliparous-primiparous by time interaction after summer 2020 surge^c^	0.14 (−0.04 to 0.31)	.14	
Nulliparous-primiparous by time interaction after fall 2020 surge^c^	−0.41 (−0.81 to −0.001)	.05	

Abbreviation: NA, not applicable.

^a^ Models include participant age, race and ethnicity, parity, partnership and cohabitation, food insecurity, and recruitment site. The Desire to Avoid Pregnancy score is on a range of 0 to 4 points, with 4 indicating greater desire to avoid pregnancy.

^b^ Slope between change points can be derived by summing coefficients.

^c^ Time is treated continuously; coefficients and slopes are reported in quarters (eg, per 3 months).

Similarly, before the summer 2020 surge, DAP scores declined more steeply among nulliparous and primiparous participants than multiparous participants (−0.07 point per quarter [95% CI, −0.09 to −0.05 point per quarter] vs −0.03 point per quarter [95% CI, −0.06 to −0.004 point per quarter]; *P* = .04) (Table 3 and Figure 2C). DAP trajectories among nulliparous and primiparous participants reversed after the summer surge to 0.11 point per quarter (95% CI, 0.02 to 0.21 point per quarter; change in slope, *P* < .001), whereas they attenuated slightly among multiparous participants to 0.01 point per quarter (95% CI, −0.13 to 0.15 point per quarter; no significant change in slope).

## Discussion

In this longitudinal cohort study in the US Southwest, the summer 2020 surge in COVID-19 cases was associated with a substantial attenuation in a prior time trend toward greater desire for pregnancy. The steady decline in preference to avoid pregnancy before summer 2020 was observed across sociodemographic groups. However, although the surge did not substantively alter pregnancy desires among women aged 25 to 34 years and multiparous women, it significantly changed trajectories among those aged 15 to 24 years, nulliparous, or primiparous, marking a shift toward greater desire to avoid pregnancy.

The steady decline in DAP scores before the pandemic likely reflects an age association; as reproductive-aged individuals get older, they approach a life stage at which they may want to raise a child. This reduction in desire to avoid pregnancy over time was more marked for those who would be having a first or second child than for those who would be having a third or higher order child. These differences aside, the reversing in this age trend associated with the first COVID-19 case surge bolsters early evidence suggesting that, early in the pandemic, people’s openness toward the prospect of pregnancy was reduced.^6,7,8,9,10,11,12,13^ Notably, in the US Southwest, COVID-19 case rates remained low around April 2020, when the shelter-in-place mandate went into effect, and the curtailing of the DAP score decline occurred 3 months later during the first surge in COVID-19 cases in the region. This timing suggests individuals’ pregnancy preferences may have been associated more with increasing regional COVID-19 case rates than shelter-in-place mandates enacted in response to high case rates nationally.

Counter to our hypotheses, during the large fall 2020 case surge regionally and nationally, DAP scores returned to their prepandemic declining trend, rather than further increasing (although the change in slope was not significant). This trend suggests population-level changes in pregnancy preferences were short lived; as it became clear that the pandemic would be a long-standing public health emergency, individuals returned to their original trajectories. Such a trend would be consistent with fertility trends seen in reaction to other emergencies, wars, or natural disasters, during which research has found short-term declines in fertility, followed by rebounds.^25,26^ The age interaction we detected might reflect women’s greater flexibility in pregnancy preferences when they have more years to conceive following the emergency.

Evidence of reduced birth rates in late 2020 and early 2021 has emerged for the US and for the southwestern states, with a possible subsequent rebound.^27,28^ Our finding of a temporary curtailing of the pre-COVID-19 trend toward greater openness to pregnancy at the first COVID-19 surge in the Southwest is generally consistent with these rates. However, multiple factors are associated with birth rates, and not all individuals preferring to prevent or postpone childbearing were able to do so. Research has documented substantial constraints in accessing contraceptive and abortion care early in the pandemic,^4,5,29,30,31,32^ followed by implementation of novel approaches to expand access to care via telehealth, mail order, and pharmacies.^33,34^ Given that social and economic disruptive events can cause population-level increases in people’s desire to prevent or postpone pregnancy, continued implementation and evaluation of these expanded care models will be important to attain reproductive autonomy during future disruptions.

### Limitations and Strengths

Because the COVID-19 pandemic was a ubiquitous exposure, we relied on 1-sample interrupted time series, rather than including a control group and examining differential changes by exposure. We, thus, could not account for other large-scale events that could have affected pregnancy preferences in 2019 to 2021. Because of our declining sample size as participants exited the study over 2020, we had limited precision to detect significant trends in later 2020 and early 2021. As a result of the association of age with parity, we lacked precision to fully disentangle each of their independent associations. In addition, this research was in 1 geographical region (US Southwest) and focused on sexually active people aged 15 to 34 years with the capacity for pregnancy who had access to health care. Results may not be generalizable beyond these groups.

The study also has substantial strengths. Because we were already following a cohort before COVID-19 and regularly measuring their pregnancy preferences, we were able to prospectively examine individual-level trends over time both before and during the pandemic. We measured changes in peoples’ pregnancy preferences with repeated measurements over time, rather than asking people to evaluate themselves how their preferences had changed. Our use of a robust instrument to measure pregnancy preferences allowed us to capture subtle changes in preferences along a continuum.

## Conclusions

The findings of this study suggest that the COVID-19 pandemic onset was associated with a temporary stalling of a prior trend toward greater desire for pregnancy over time, particularly for people earlier in their reproductive lives. Expanded contraceptive and abortion care models, such as pharmacy access, telemedicine, and mail order, will be important to reproductive autonomy during future disruptions to medical care access.

## Notes

Supplement. **eFigure.** Centers for Disease Control and Prevention Tracking of the Trend in Number and Seven-Day Average of Positive COVID-19 Cases in Arizona, March 15, 2020-March 15, 2021 **eTable.** Desire to Avoid Pregnancy (DAP) Scale Sample Item Frequencies at Baseline (N=627), Selected From 14 Items
