# Supplementary material for: Comparison of Pregnancy Preferences Preceding vs Year 1 of the COVID-19 Pandemic
Source: JAMA Netw Open. 2022 Jul 5;5(7):e2220093. doi: 10.1001/jamanetworkopen.2022.20093 (PMC9257576; doi:10.1001/jamanetworkopen.2022.20093)
Supplement: Supplement. — eFigure. Centers for Disease Control and Prevention Tracking of the Trend in Number and Seven-Day Average of Positive COVID-19 Cases in Arizona, March 15, 2020-March 15, 2021 eTable. Desire to Avoid Pregnancy (DAP) Scale Sample Item Frequencies at Baseline (N=627), Selected From 14 Items [file jamanetwopen-e2220093-s001.pdf]

Supplemental Online Content

Rocca CH, Parra M, Muñoz I, Foster DG, Boscardin WJ, Ralph LJ. Comparison of pregnancy preferences preceding vs year 1 of the COVID-19 pandemic. *JAMA Netw Open*. 2022;5(7):e2220093. doi:10.1001/jamanetworkopen.2022.20093

eFigure. Centers for Disease Control and Prevention Tracking of the Trend in Number and Seven-Day Average of Positive COVID-19 Cases in Arizona, March 15, 2020-March 15, 2021

eTable. Desire to Avoid Pregnancy (DAP) Scale Sample Item Frequencies at Baseline (N=627), Selected From 14 Items

This supplemental material has been provided by the authors to give readers additional information about their work.

© 2022 Rocca CH et al. *JAMA Network Open.*

eFigure. Centers for Disease Control and Prevention Tracking of the Trend in Number and Seven-Day Average of Positive COVID-19 Cases in Arizona, March 15, 2020-March 15, 2021

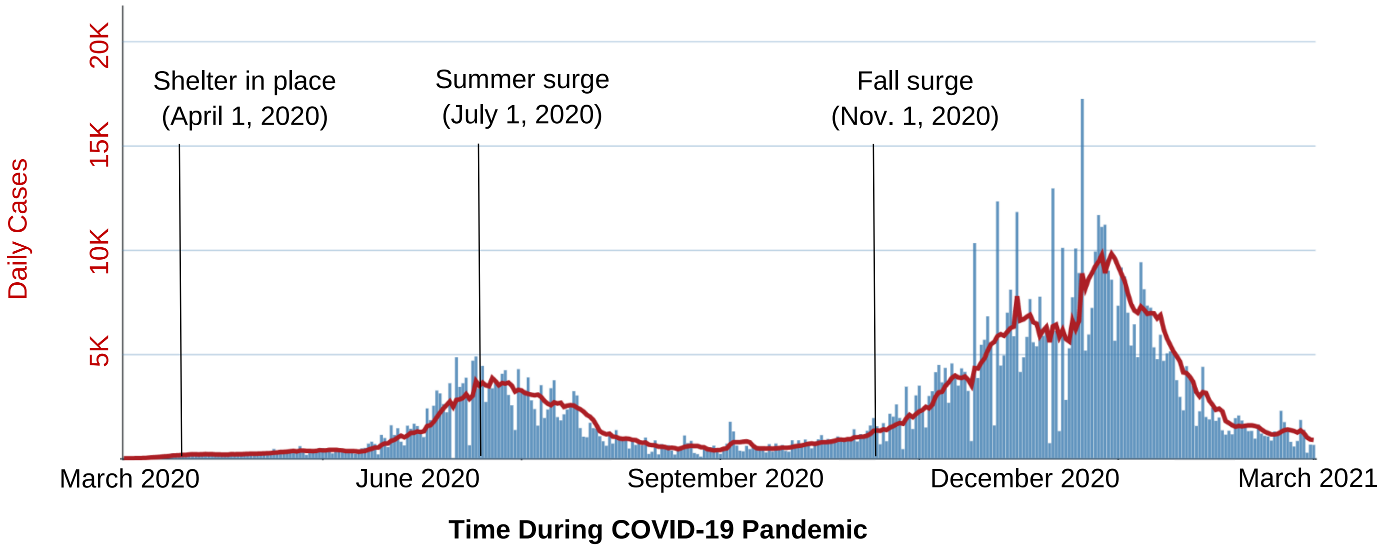

Adapted from: Centers for Disease Control and Prevention. COVID Data Tracker. https://covid.cdc.gov/covid-data-tracker/#datatracker-home. Published 2021. Accessed October 1, 2021.

© 2022 Rocca CH et al. *JAMA Network Open.*

eTable. Desire to Avoid Pregnancy (DAP) Scale Sample Item Frequencies at Baseline (N=627), Selected From 14 Items

	**N (%)**
**I want to have a baby within the next year.**	
Strongly agree	86 (13.7)
Agree	63 (10.1)
Neither agree nor disagree	92 (14.7)
Disagree	159 (25.4)
Strongly disagree	227 (36.2)
**It would be the end of the world for me to have the baby in the next year.**	
Strongly agree	70 (11.2)
Agree	53 (8.5)
Neither agree nor disagree	120 (19.1)
Disagree	183 (29.2)
Strongly disagree	201 (32.1)
**Thinking about becoming pregnant in the next 3 months makes me feel excited.**	
Strongly agree	68 (10.9)
Agree	64 (10.2)
Neither agree nor disagree	139 (22.2)
Disagree	136 (21.7)
Strongly disagree	220 (35.1)
**If I had a baby in the next year, it would be hard for me to manage raising the child.**	
Strongly agree	108 (17.2)
Agree	94 (15.0)
Neither agree nor disagree	152 (24.2)
Disagree	158 (25.2)
Strongly disagree	115 (18.3)

© 2022 Rocca CH et al. *JAMA Network Open.*
